# Supplementary material for: Therapeutic targeting of 15-PGDH in murine pulmonary fibrosis
Source: Sci Rep. 2020 Jul 15;10:11657. doi: 10.1038/s41598-020-68336-0 (PMC7363833; doi:10.1038/s41598-020-68336-0)
Supplement: Supplementary file 1 — Supplementary file1 (PDF 817 kb) [file 41598_2020_68336_MOESM1_ESM.pdf]

Therapeutic Targeting of 15-PGDH in Murine Pulmonary Fibrosis

Julianne N.P. Smith, Matthew D. Witkin, Alvin P. Jogasuria, Kelsey F. Christo, Thomas M. Raffay, Sanford D. Markowitz‡, and Amar B. Desai*

Department of Medicine, Case Western Reserve University, Cleveland OH 44106 USA

‡Denotes co-corresponding author

*Correspondence:

Amar B. Desai

Department of Medicine

Case Western Reserve University

Abd10@case.edu

Running title: PGDHi therapy for pulmonary fibrosis

Supplementary Information: Four supplemental figures accompany this paper

Supplemental Figure Legends:

Supplementary Figure 1: PGDHi results in a trend towards mitigated pulmonary inflammation following intratracheal installation of bleomycin. (**A**) Schematic depicting intratracheal administration of 2mg/kg Bleomycin to 8wk old C57BL/6 female mice with subsequent PGDHi therapy (5mg/kg (+)SW033291, twice per day) and sacrifice at day 14. (**B**) Inflammatory factors CXCL1 and TNFα were measured in lung lysates of healthy naïve control (-), and Veh- and PGDHi-treated mice 14 days post-bleomycin administration by multiplex ELISA. Individual data points and mean±SEM are depicted; n=2-4 mice/group. (**C**) Representative images of Masson's trichrome-stained lung sections from healthy naïve control, and vehicle- and PGDHi-treated mice 14 days post-intratracheal bleomycin administration. 20X, scale bars represent 200µm.

Supplementary Figure 2: PGDHi leads to improvements in overall health and survival of mice following intratracheal instillation of bleomycin. (**A**) Schematic depicting intratracheal administration of 2mg/kg Bleomycin to 8wk old C57BL/6 female mice with subsequent PGDHi therapy (5mg/kg (+)SW033291, twice per day) through day 35. (**B**) Body weight was measured for thirty-five days post-bleomycin administration as a percentage of day 0 weight for each mouse, treated as indicated. Mean±SEM is depicted. n=10 mice/group at the onset of the study, and n=5-7 mice/group at the end of the study. A two-way ANOVA with post-hoc Sidak's multiple comparisons test was used to compare vehicle- versus PGDHi-treated mice. (**C**) Kaplan-Meier survival curve following intratracheal bleomycin administration. N=25 mice per group.

Supplementary Figure 3: Pulmonary 15-PGDH expression is maintained following bleomycin exposure. Representative images of 15-PGDH staining in lung sections from vehicle- and PGDHi-treated mice at days 7 and 35 post-bleomycin exposure.

Supplementary Figure 4: PGDHi reduces fibrosis 35 days post-intratracheal instillation of bleomycin. (**A**) Schematic depicting intratracheal administration of 2mg/kg Bleomycin to 8wk old C57BL/6 female mice with subsequent PGDHi therapy (5mg/kg (+)SW033291, twice per day) through day 35. (**B**) Lung hydroxyproline levels were measured in tissue lysates from healthy naïve control (-), and Veh- and PGDHi-treated mice 35 days post-bleomycin administration. Individual data points and mean±SEM are depicted; n=15-20 experimental mice/group. An ordinary one-way ANOVA with post-hoc Tukey multiple comparisons test was used to compare groups. (**C**) Representative images of Masson's trichrome and elastin-stained lung sections from healthy naïve control, and vehicle- and PGDHi-treated mice 35 days post-intratracheal bleomycin administration. 20X, scale bars represent 200μm. *P=0.01, ***P=0.0004 for indicated comparisons.

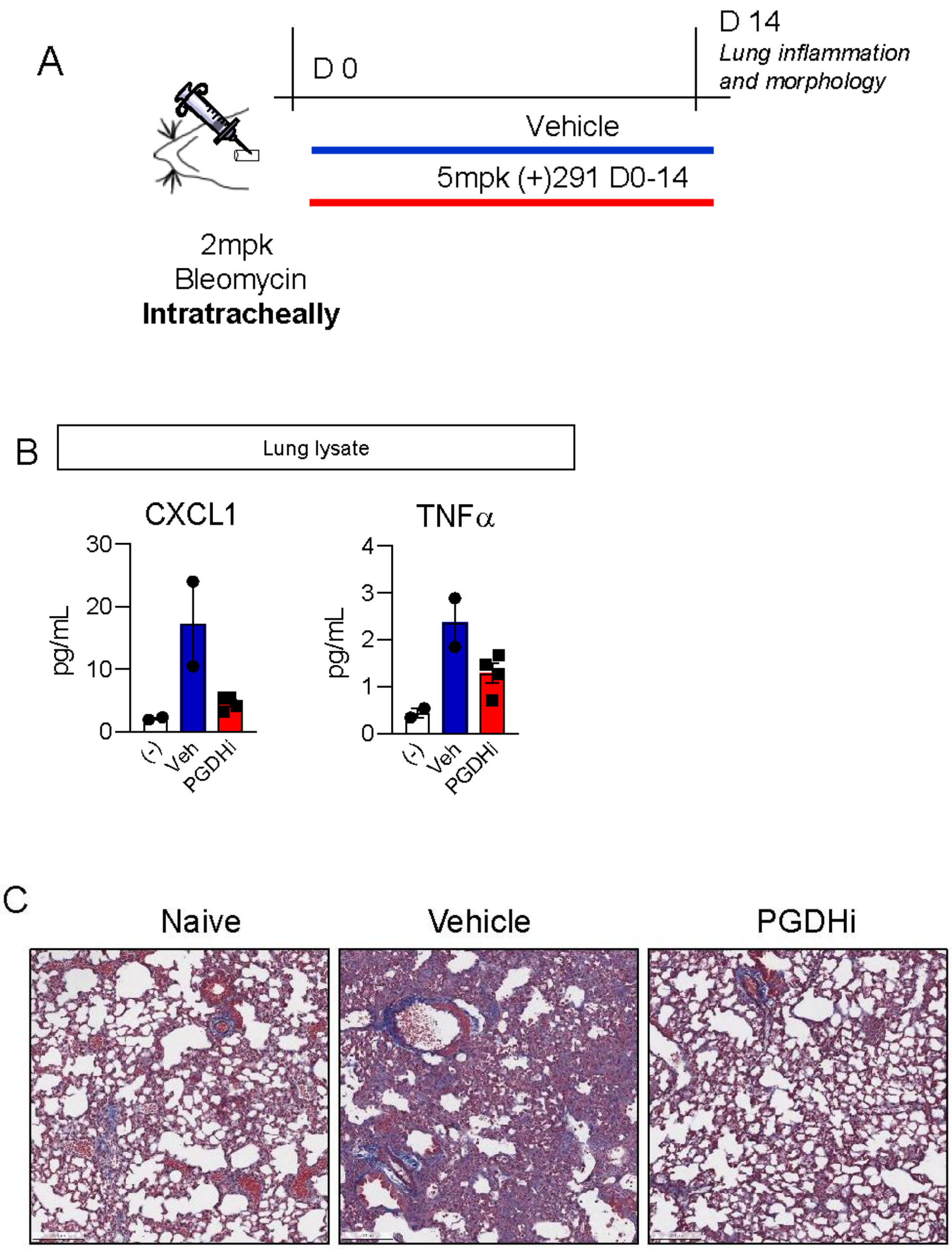

A
D 0
D 14
Lung inflammation
and morphology
Vehicle
5mpk (+)291 D0-14
2mpk
Bleomycin
Intratracheally
B
Lung lysate
CXCL1
TNFα
pg/mL
30
20
10
0
4
3
2
1
0
(-)
Veh
PGDHi
C
Naive
Vehicle
PGDHi

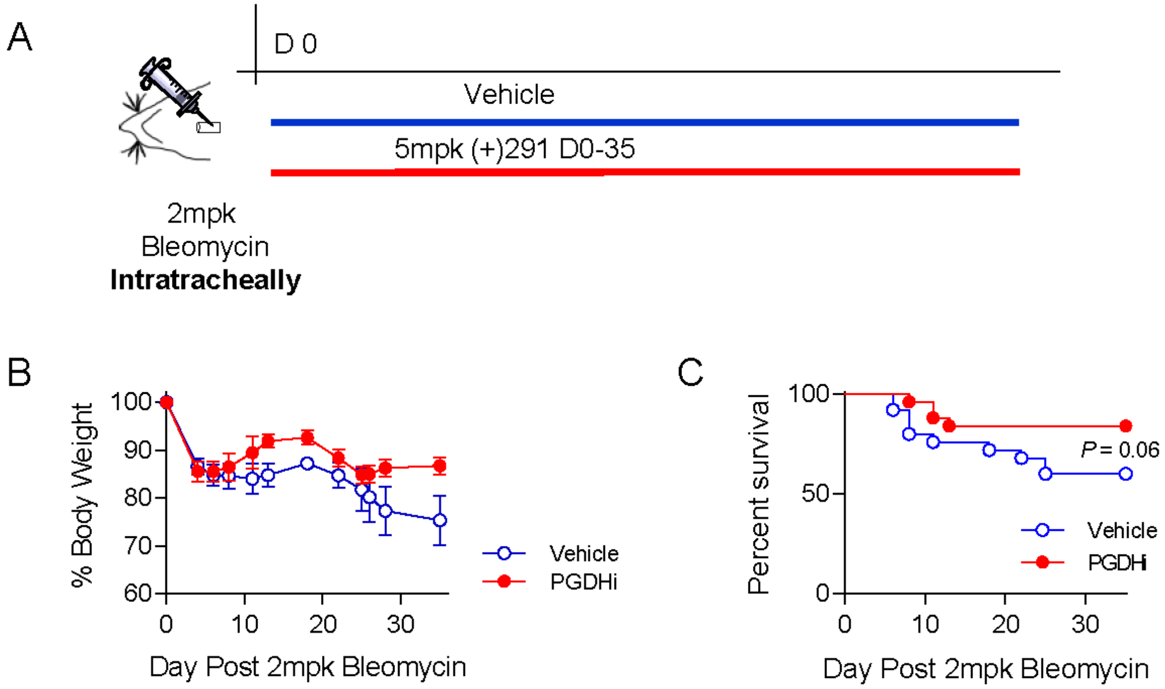
A
D 0
Vehicle
5mpk (+)291 D0-35
2mpk
Bleomycin
Intratracheally
B
% Body Weight
Day Post 2mpk Bleomycin
Vehicle
PGDHi
C
Percent survival
P = 0.06
Vehicle
PGDHi
Day Post 2mpk Bleomycin

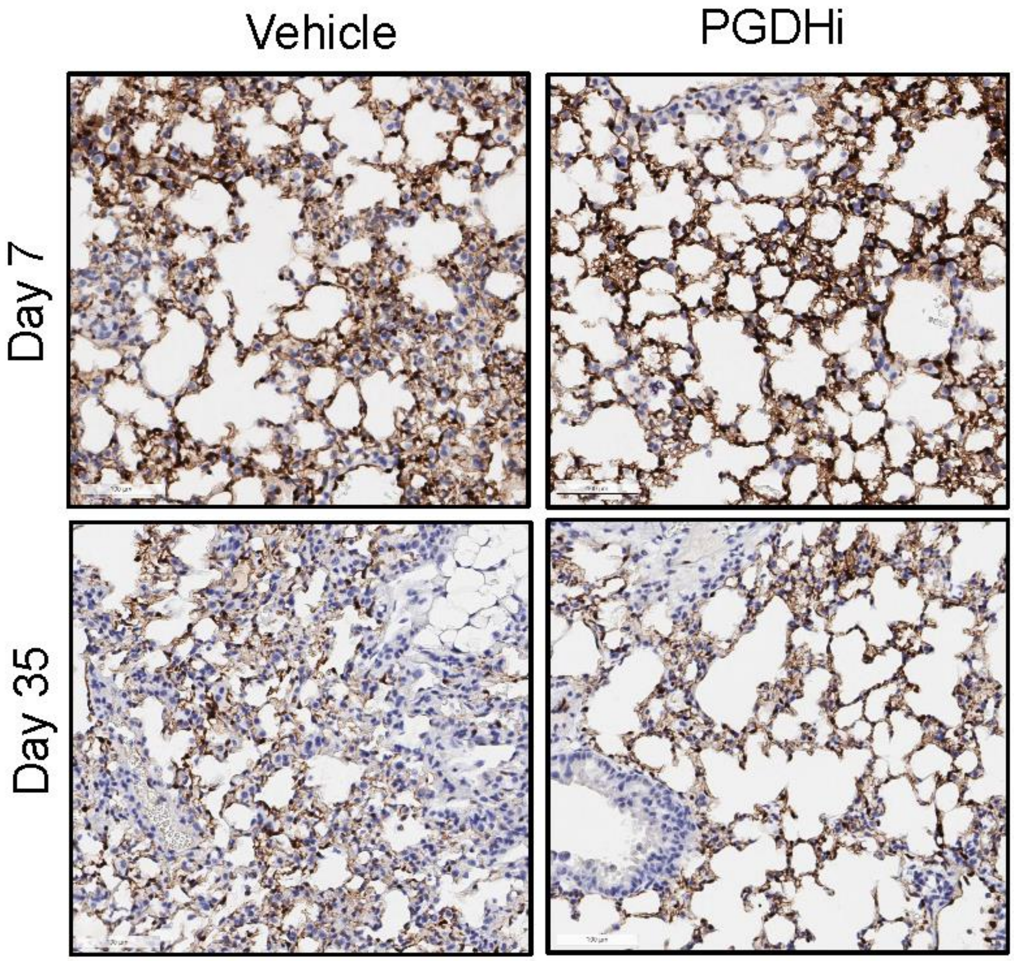
Vehicle
PGDHi
Day 7
Day 35

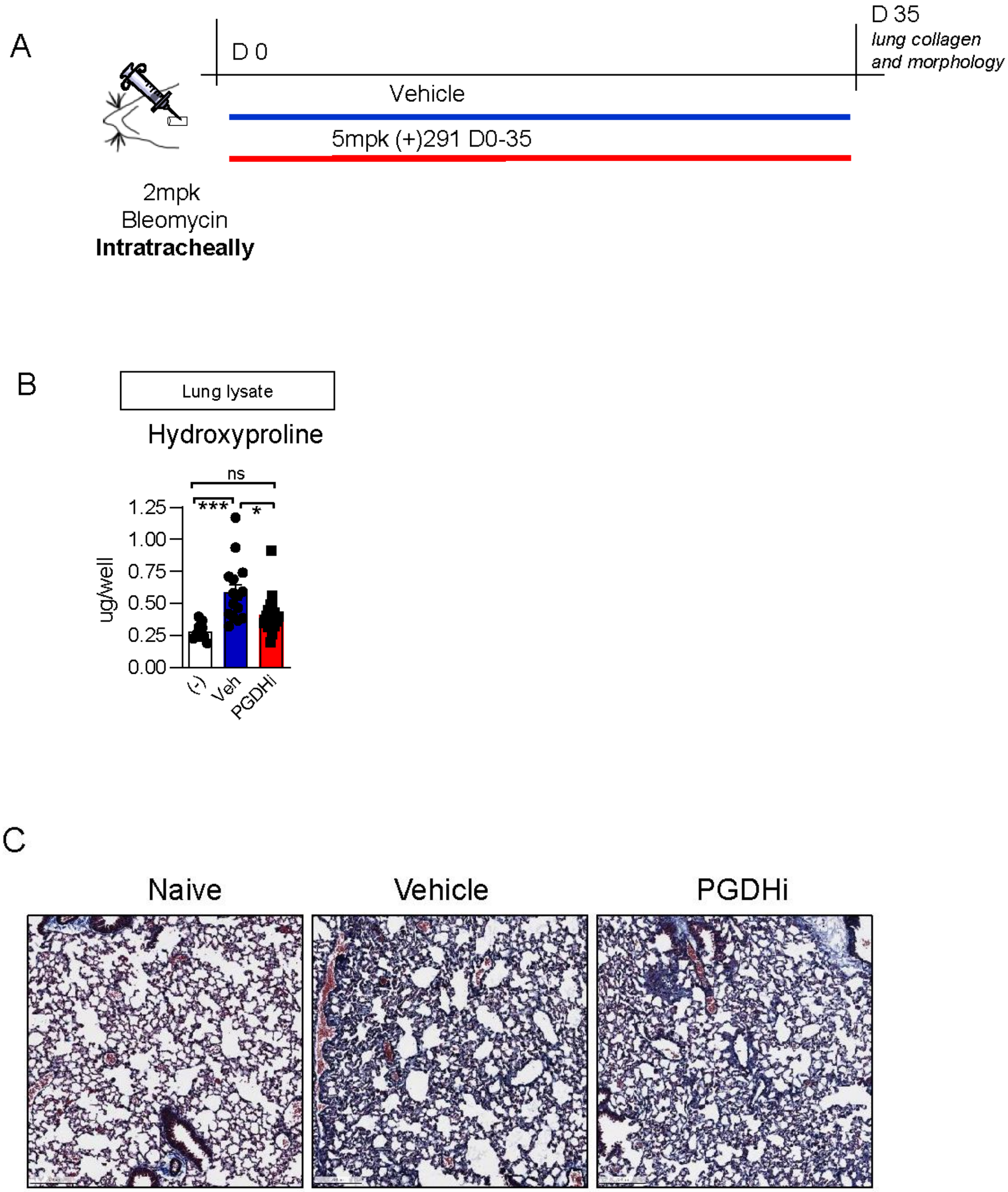
A
D 0
D 35
lung collagen
and morphology
Vehicle
5mpk (+)291 D0-35
2mpk
Bleomycin
Intratracheally
B
Lung lysate
Hydroxyproline
ns
***
*
1.25
1.00
0.75
0.50
0.25
0.00
ug/well
(-)
Veh
PGDHi
C
Naive
Vehicle
PGDHi
